# Exploring the Role of Tamarind Seed Polysaccharides in Modulating the Structural, Digestive, and Emulsion Stability Properties of Waxy Corn Starch Composites

**DOI:** 10.3390/foods14234152

**Published:** 2025-12-03

**Authors:** Xiangyu Ya, Yongshuai Ma, Zibo Song, Yongli Jiang, Chaofan Guo, Junjie Yi

**Affiliations:** 1 Faculty of Food Science and Engineering, Kunming University of Science and Technology, Kunming 650500, China; 2 Key Laboratory of Plateau Characteristic Prepared Food in Yunnan Province, Kunming 650500, China; 3 International Green Food Processing Research and Development Center of Kunming City, Kunming 650500, China; 4 Yunnan Engineering Research Center for Fruit & Vegetable Products, Kunming 650500, China; 5 Yunnan Maoduoli Key Laboratory of Applied Technology for Special Forest Fruits, Yuxi 653100, China; 6 Yunnan Maoduoli Group Food Co., Ltd., Yuxi 653100, China

**Keywords:** waxy maize starch, tamarind seed polysaccharide, O/W emulsions, in vitro digestion

## Abstract

This study investigated the effects of tamarind seed polysaccharide (TSP) on the structural characteristics, digestibility, and emulsifying properties of waxy maize starch (WMS), as well as their interaction mechanisms. WMS-TSP complexes were prepared via complexes to improve starch’s physical and functional properties. Native WMS showed smooth spherical granules, while WMS-TSP samples formed freeze-drying-induced honeycomb structures (~200–250 μm). In vitro digestion indicated that WMS-TSP systems (5–15%) reduced RDS by 20.1–24.11% relative to native WMS (41% ± SD), suggesting a potential to attenuate postprandial glycemic responses. Fourier-transform infrared (FT-IR) spectroscopy revealed that TSP interacted with WMS mainly through non-covalent bonds such as hydrogen bonding, while influencing the degree of crystallinity without generating new crystalline polymorphs. In corn oil-based emulsions, the WMS-TSP composites showed strong viscoelastic behavior, with elevated storage (G′) and loss (G″) moduli, together with improved storage stability. These findings highlight the synergistic potential of WMS and TSP in enhancing the functionality of starch-based systems and provide insights into the role of polysaccharides in food structure and digestion regulation.

## 1. Introduction

Over the past decade, emulsions have garnered significant research attention due to their exceptional stability against droplet flocculation [1]. The increasing demand for clean label foods has encouraged the food industry to explore functional ingredients from natural sources [2]. Traditionally, many emulsifiers used in the food industry have been chemically synthesized (such as surfactants) or derived from animal sources (such as milk, eggs, or meat) [3]. Recently, there has been growing interest in plant-based emulsifiers, as they are considered more sustainable and label-friendly. Many plant-based emulsifiers are amphiphilic molecules capable of adsorbing at the oil–water interface [4]. While these molecular emulsifiers are effective in forming small droplets during homogenization, they are less efficient in preventing droplet coalescence during storage. Consequently, there is a keen interest in using colloidal complexes as emulsifiers, as they are more effective in inhibiting droplet coalescence. Therefore, the development of plant-based colloidal complexes capable of forming and stabilizing food emulsions has become a crucial area of research.

Starch, as a rich and cost-effective plant-based material, finds extensive application in the formation and stabilization of food emulsions [5]. Colloidal complexes based on starch serve as effective emulsifiers, forming protective layers at the oil–water interface to enhance the physical stability of the emulsion [6]. These complexes can be tailored through physical, chemical, or enzymatic modifications to adjust their size [7], morphology, and surface properties to meet various application needs. Starch primarily consists of amylose and amylopectin, with amylose exhibiting stronger complexation ability with guest molecules due to its longer chains. Despite the stronger complexation ability of high-amylose starches, waxy maize starch was chosen due to its industrial relevance, high paste stability, and minimal retrogradation, which allow clearer assessment of TSP-induced structural modifications. In addition, starch can form composite complexes with specific polysaccharides, which serve as effective stabilizers in starch-based emulsions. Modified starch with polysaccharides has been demonstrated as an effective and practical approach to enhance gelatinization, thermal, rheological, and gelation properties by regulating molecular interactions [8]. Notably, Yang et al. [9] found that *Tremella fuciformis polysaccharide* (TFP) incorporation significantly improved the gelatinization and cooking characteristics of potato starch through structural modification. Furthermore, Zhou et al. [10] revealed that laminaria polysaccharide could effectively inhibit wheat starch retrogradation by altering starch molecular architecture. Additionally, a recent study by Shi et al. [11] demonstrated that Ginkgo biloba non-starch polysaccharides enhanced the thermal stability and gel strength of oat starch without affecting its crystalline structure or molecular conformation. Thus, the pursuit of highly efficient plant-based colloidal complexes for the stabilization of food emulsions continues to be a matter of significant relevance and importance in this field.

Tamarind Seed Polysaccharide (TSP) is a typical xyloglucan, composed of galactose, xylose, and glucose in an approximate ratio of 1:2:3 [12]. TSP is regarded as an efficient dietary polysaccharide and is deemed to be the most potential one among the new edible colloids, having the prospect of competing with the existing commercial water-soluble polysaccharides and demonstrating extensive application prospects in the food industry. TSP exhibits excellent water solubility, presenting a rigid extended structure in aqueous solution without random coil conformation. Due to intermolecular interactions among TSP molecules, supramolecular aggregates can form even in dilute solutions, with the degree of aggregation depending on the extent of side-chain substitution [13]. De-galactosylated TSP displays increased molecular aggregation, resulting in the formation of microgels in dilute solutions. TSP has gained extensive applications across various sectors of the food industry due to its advantageous properties, which include high viscosity, broad pH tolerance, non-carcinogenicity, mucosal adhesion characteristics, biocompatibility, and significant drug encapsulation capacity. Research on the impact on starch indicates that TSP may interact with starch granules under high-temperature conditions, enhancing the water absorption rate and expansion rate of starch granules. This interaction may alter the structure of starch granules and inhibit gelatinization by promoting easier water absorption and expansion [14]. Addition of TSP results in starch gels after freeze–thaw treatment exhibiting enhanced stability in terms of hardness, viscoelasticity, and recovery properties [15]. This phenomenon can be attributed to the hydrophilic groups inherent in the TSP structure. The presence of these hydrophilic groups facilitates the effective attraction and binding of water molecules by starch gels upon contact, thereby establishing a stable network architecture. This network not only enhances moisture retention but also restricts the mobility of water within the starch gel, consequently reducing the fraction of water that is susceptible to freezing [16]. Therefore, TSP contributes to starch modification through its unique gel-forming properties. However, the specific effects of TSP on the short-range molecular order of waxy maize starch, its in vitro digestibility characteristics, and its ability to stabilize oil-in-water emulsions have not yet been systematically elucidated.

Therefore, this study aimed to elucidate the interaction mechanisms between WMS and TSP and to determine how these interactions modulate the structural organization, digestive behavior, and emulsion stability of WMS-based composite systems. By establishing these relationships, the work seeks to deepen current understanding of starch–polysaccharide compatibility and provide a framework for designing clean-label starch composites with tailored functional attributes for modern food applications.

## 2. Materials and Methods

### 2.1. Materials

The waxy maize starch was obtained from Xintai Food Chemical Company (Shanghai, China). Its basic composition, including moisture (12.7%), ash (0.09%), protein (0.28%), and crude fat (<0.1%), was analyzed in our laboratory using AOAC standard methods. Corn oil was obtained from JinLongYu, while Nile red and Nile blue A were purchased from Aladdin (Shanghai, China). The tamarind seed polysaccharide, obtained from Yunnan Maoduoli, (Yuxi, China), has a purity greater than 90.1%, with a protein content of 1.1%, moisture content at 8.8%, ash content of 0.3% and fat <0.2%. The structural features of TSP are shown in Figure S1. The purity of all other chemical reagents is at a minimum analytical reagent grade or higher.

### 2.2. Synthesis of WMS-TSP Complexes

The WMS-TSP complex was prepared using the existing ethanol precipitation method, with modifications and optimizations applied to the process [8]. To synthesize WMS-TSP complexes, WMS was dispersed in deionized water at a 1:50 (*w*/*v*) ratio and heated with continuous stirring in a 95 °C water bath for 40 min, a condition known to induce complete gelatinization of WMS. Subsequently, TSP was incorporated at 5%, 10%, and 15% (*w*/*w*, relative to WMS), a range selected to enable cooperative structuring between WMS and TSP rather than simple additive mixing, while avoiding the viscosity-related processing issues observed at higher TSP levels. Following this, the mixture was heated to a temperature of 95 °C and stirred for an additional duration of 20 min. Upon completion of the thermal treatment, the WMS-TSP composite solution was cooled to 25 °C and subsequently introduced, via controlled dropwise addition at 1.0 mL/min, into a fourfold volume of anhydrous ethanol (1:4, *v*/*v*; 25 °C) to induce phase separation through antisolvent precipitation. Centrifuge the mixture at 3500 revolutions per minute for a duration of 15 min. The collected precipitate was filtered, washed three times with anhydrous ethanol, frozen at −70 °C, and subsequently lyophilized under vacuum to remove residual water and ethanol, yielding the WMS-TSP complexes. Throughout preparation, the dispersion exhibited a native pH of 6.3 ± 0.1, and no pH adjustment was applied to prevent altering WMS-TSP associative behavior.

### 2.3. Fabrication of Emulsions Stabilized by WMS-TSP Complexes

The WMS-TSP complexes containing 10% TSP were separately dispersed in aqueous solutions with varying mass concentrations of 0.5%, 1.0%, 1.5%, and 2.0% (*w*/*v*) using deionized water as the dispersion medium. Emulsions were prepared at a corn oil volume fraction of ϕ = 16.7% (*v*/*v*), corresponding to an oil-to-water ratio of 1:5 (*v*/*v*). Using an XM-1200T ultrasonic cell disruptor (Kunshan, China, 20 Khz). Ultrasonication was performed at 400 W and 60% amplitude using a 12 mm titanium probe in pulsed mode (5 s on/3 s off) for a total of 2 min, while maintaining the temperature below 30 °C with an ice-water bath. The resulting O/W emulsions were prepared at particle concentrations of 0.5%, 1.0%, 1.5%, and 2.0% (*w*/*v*) and transferred into 4 mL transparent vials (Ø 15 mm × 45 mm) in triplicate for subsequent observation.

### 2.4. Characteristics of WMS-TSP Complexes

#### 2.4.1. Scanning Electron Microscopy (SEM)

The microstructure of WMS-TSP complexes (c = 5–15%) was characterized using an Apreo 2S scanning electron microscope (Thermo Fisher Scientific, Waltham, MA, USA). Prior to observation, the starch complexes were fixed onto tape and coated with a thin layer of gold.

#### 2.4.2. Transmission Electron Microscopy (TEM)

The morphology of WMS-TSP complexes was evaluated using a transmission electron microscope (TEM; JEM-F200, JEOL, Tokyo, Japan). Before observation, the sample should be suspended in ethanol without water at a concentration of 1 mg/mL. Subsequently, it should undergo ultrasonic treatment for 15 min to ensure uniform dispersion of the sample. Following this treatment, the suspension is deposited onto a copper grid coated with carbon film. After the ethanol evaporated, the samples were placed in the TEM sample chamber for morphological observation and imaging of the WMS-TSP complexes.

#### 2.4.3. Fourier-Transform Infrared Spectroscopy (FT-IR) Analysis

Potassium bromide (KBr) was incorporated into the WMS-TSP composite system at a mass ratio of 1:50 and thoroughly ground. Following this, the powder samples were compressed into cylindrical pellets using a hydraulic press. Spectral measurements were performed using a Nicolet 6700 FTIR spectrometer (Thermo Fisher Scientific) within the mid-infrared range of 4000 to 400 cm^−1^. The resolution was set at 4 cm^−1^, and each sample underwent 32 scans. The spectra within 900–1200 cm^−1^ were smoothed and deconvolved using PeakFit 4.12. The absorption bands at 1047, 1022, and 995 cm^−1^ were quantified by measuring the peak heights above the local baseline after baseline correction.

#### 2.4.4. X-Ray Diffraction (XRD)

The WMS-TSP complexes (0–15% TSP) were analyzed by X-ray diffraction using a diffractometer equipped with Cu Kα radiation (λ = 0.1542 nm) operated at 40 kV and 100 mA. Diffraction patterns were collected over a 2θ range of 4–45° with a step size of 0.02° and a scan speed of 1° min^−1^. Relative crystallinity (RC) was quantified using Jade 6.5 (Materials Data Inc., Livermore, CA, USA) according to Equation (1):

(1) $$x_{c}\;=\;\frac{A_{c}}{A_{c}\;+\;A_{a}}$$

In Equation (1), A*_c_* represented the areas of crystalline region; and A*_a_* represented the areas of amorphous region.

#### 2.4.5. Particle Size Measurement

The distribution of WMS-TSPs particle sizes were determined by a ZSU3100 nanoparticle size (Malvern Panalytical Ltd., Malvern, UK). following the previously reported procedure [17]. Lyophilized powders (0.1% *w*/*v*) were dispersed in deionized water, mildly sonicated, and centrifuged to remove coarse aggregates. Measurements were conducted at 25 °C using water as the dispersant (RI = 1.33). Volume-based D_10_, D_50_, and D_90_ values were obtained and expressed in μm.

#### 2.4.6. Three-Phase Contact Angle Measurement

The three-phase contact angle (θ) was measured by an OCA-20 optical contact angle goniometer (DataPhysics Instruments, Filderstadt, Germany). Starch powders were compressed into tablets (13.0 mm in diameter, 2.0 mm in thickness) and completely immersed in corn oil to allow the solid–oil interface to reach equilibrium. While submerged, a 15 μL droplet of distilled water was gently deposited onto the tablet surface. After equilibrating for 1.0 min, the droplet profile was recorded using a high-speed camera, and θ was calculated based on the Laplace–Young fitting.

#### 2.4.7. In Vitro Digestibility

Starch digestion was carried out following the method of Englyst et al. [18] with some modifications, which focuses on intestinal enzymatic hydrolysis and is commonly used for determining rapidly digestible starch (RDS), slowly digestible starch (SDS), and resistant starch (RS). The digestion process was performed by mixing 100 mg of each sample with 5 mL of phosphate buffer (pH 5.2) containing α-amylase (260 U/mL) and starch glucosidase (9 U/mL), followed by incubation with digesting by shaking at 37 °C. At 0, 20, 60, 90, 120, and 150 min, 0.2 mL samples were withdrawn and combined with 1.8 mL of absolute ethanol, followed by centrifugation at 10,000 rpm for 5 min to isolate the supernatant. Glucose concentration was determined using a glucose oxidase–peroxidase assay kit (Nanjing, China). The contents of RDS, SDS, and RS were calculated using the following formulas:

(2) $$\mathrm{RDS}\;(\%)\;=\;\frac{G20-G0}{\mathrm{TS}}\;\times \;0.9\;\times \;100$$

(3) $$\mathrm{SDS}\;(\%)=\;\frac{G120-G20}{\mathrm{TS}}\;\times \;0.9\;\times \;100$$

(4) $$\mathrm{RS}\;(\%)=\;\frac{\mathrm{TS}-\mathrm{RDS}-\mathrm{SDS}}{\mathrm{TS}}\;\times \;0.9\;\times \;100$$

G0 was the free glucose mass before hydrolysis. G20 and G120 rep resented the glucose content released after 20 and 120 min of hydrolysis, respectively. TS was a total dry mass of WMS and WMS-TSP.

#### 2.4.8. Kinetic Modeling of In Vitro Starch Digestion

All digestion profiles of WMS and WMS-TSP complexes were analyzed using a first-order kinetic model (LOS) to quantify the hydrolysis behavior. The digestion curves (*Ct* vs. *t*) were fitted to the following Equation (5):

Ct = C∞ (1 − e^−kt^) (5)

where *Ct* is the starch hydrolyzed at time *t*, *C∞* represents the equilibrium hydrolysis extent, and *k* is the digestion rate constant. Nonlinear least-squares regression was performed using Python 3.10 (SciPy1.11), and the fitted parameters (*C∞*, *k*) together with the coefficient of determination (R^2^) were obtained for each sample. Kinetic fitting was based on the mean digestion curves derived from independent replicates.

### 2.5. Characteristics of WMS-TSP Complexes Emulsions

#### 2.5.1. Storage Stability

The emulsification index (EI) was used to evaluate the storage stability of the emulsions. Photographs of the freshly prepared emulsions were taken on days 0, 30, 60, and 90 to observe changes over time. The initial emulsion height (H_0_) and the height of the remaining stable emulsion layer after standing (Hₜ) were recorded, and the emulsification index (EI) was determined using the following formula:

(6) $$\mathrm{EI}\;\left(\%\right)\;=\;\frac{H_{t}}{H_{0}}\;\times \;100$$

where H_0_ = initial emulsion height; and Hₜ = stable emulsion height after storage.

#### 2.5.2. Microstructure Observation

We employed the methodology established in previous studies [19], freshly preparing emulsions that were simultaneously stained with Nile Blue A and Nile Red dyes. The microstructures within these emulsions were examined using a confocal laser scanning microscope (FV1200, OLYMPUS, Tokyo, Japan). Fluorescent images were captured under excitation wavelengths of 543 nm and 633 nm.

#### 2.5.3. Measuring Rheology

The rheological Characteristics of emulsions stabilised with WMS-TSP complexes were determined with reference to previously reported literature, with minor modifications [20]. The dynamic rheological characteristics of emulsions stabilized by WMS-TSP complexes were carried out using an Anton Paar MCR 102 rheometer (Graz, Austria). A parallel plate geometry PU40 SR3009 (diameter 40 mm) with a probe gap of 1.00 mm was employed. A solvent trap was used during all measurements, and the tests were performed under controlled temperature and humidity to effectively limit sample evaporation. Prior to testing, samples were equilibrated at 25 °C for 5 min. The linear viscoelastic region (LVR) was first determined through an oscillatory amplitude sweep at 1 Hz, with strain amplitudes varied from 0.1 to 100 during which the elastic modulus (G′) was monitored as a function of strain. The strain at which G′ deviated by ≤5% from its plateau value was taken as the LVR limit, and all samples exhibited a stable G′ plateau between approximately 0.01% and 1% strain. Oscillatory frequency sweeps (0.1–10 Hz) were conducted at a constant strain amplitude of 0.01, which was confirmed to fall well within the LVR for all samples. Both the elastic modulus (G’) and the loss modulus (G”) were measured. Furthermore, the relationship between shear rate (0.1–100 s^−1^) and apparent viscosity was evaluated.

#### 2.5.4. Droplet Size and Zeta Potential of Emulsion

The droplet size distribution of the emulsions was analyzed using a Rise-2008 laser particle size analyzer (Jinan Rise Science & Technology Co., Ltd., Jinan, China) after 1 day of storage at room temperature. Prior to measurement, samples were gently diluted 1:100 (*v*/*v*) with deionized water, and the parameters D_10_, D_50_, D_90_, and Dav were recorded. Zeta potential was measured using a Malvern Zetasizer Nano ZS90 (Malvern Instruments Ltd., Worcestershire, UK) following a 1:100 (*v*/*v*) dilution. All measurements were performed at 25 °C.

### 2.6. Statistical Analysis

All data were analyzed using SPSS statistical software (version 24) and presented as mean ± standard deviation. Graphs analyses were generated using OriginPro 2021 software (OriginLab Corporation, Northampton, MA, USA). Statistical analyses were performed using one-way ANOVA followed by Tukey’s test, with significance set at *p* < 0.05. Each experiment was conducted in triplicate (*n* = 3), and exact *p*-values are reported in the Section 3 Results.

## 3. Results and Discussion

### 3.1. Characterization of WMS-TSP Complexes

#### 3.1.1. SEM Analysis

**Figure 1:**
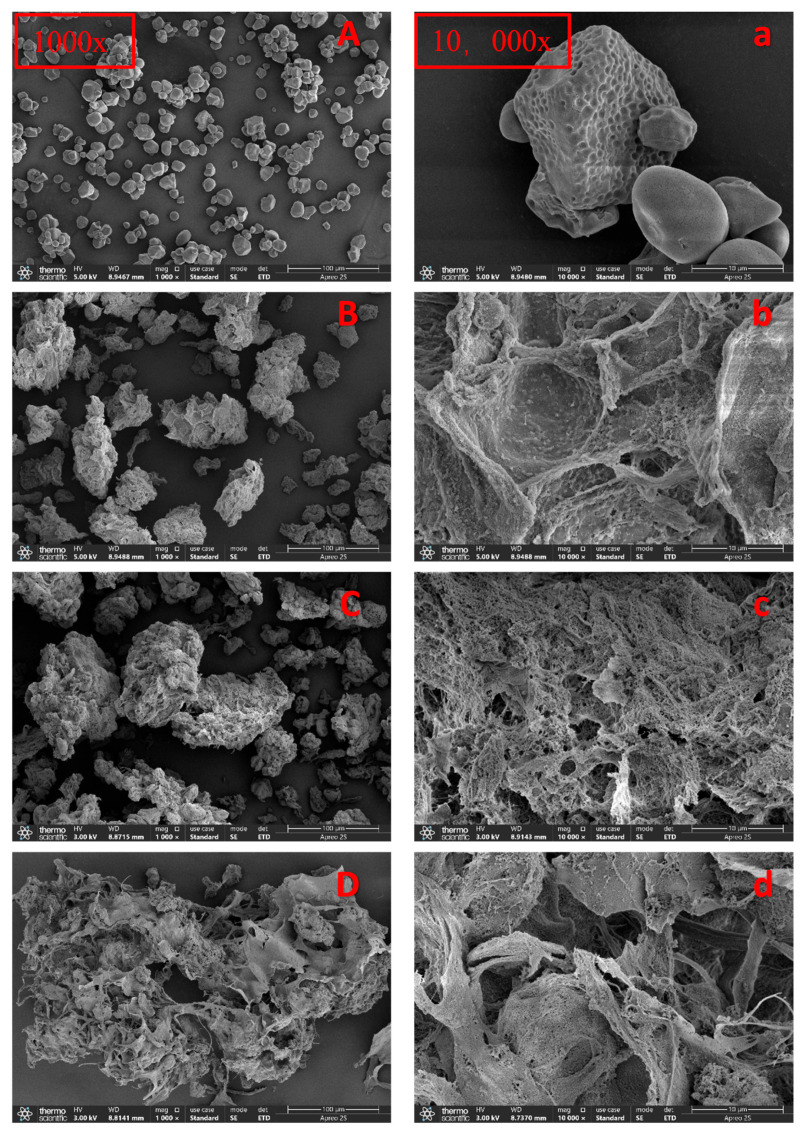
SEM photomicrographs of waxy corn starch and WMS–TSP complexes at two magnifications. (**A**–**D**) Images taken at 1000× magnification; (**a**–**d**) corresponding images taken at 10,000× magnification. (**A**,**a**) Native waxy corn starch; (**B**,**b**) WMS-TSP complex with 5% TSP; (**C**,**c**) WMS-TSP complex with 10% TSP; (**D**,**d**) WMS-TSP complex with 15% TSP.

Scanning electron microscopy was used to observe the morphological changes in starch treated with different concentrations of TSP. The complexes of WMS are predominantly round or oval, with a few irregularly shaped ones, consistent with previous reports, as shown in Figure 1A. The WMS-TSP complexes with different TSP concentrations exhibited significant differences in their microstructures (Figure 1). The WMS-TSP complex exhibited a honeycomb-like architecture resulting from moisture segregation during lyophilization which is a freeze-drying artifact rather than the hydrated microstructure present in emulsion or digestion conditions. Analogous structural features were observed in the deacetylated chitosan-sugar beet-derived betaine corn starch gel composite system, demonstrating similar phase separation mechanisms during the freeze-drying process [21].

Elevated concentrations of TSP were observed to compromise the porous architecture, primarily through the reorientation of amylose molecules and concomitant moisture segregation within the composite system. This resulted in the formation of wrinkles and tears on the surface of the gel network, as reported by Ren et al. [22]. Furthermore, SEM images at 10,000× magnification revealed that TSP adsorbed onto the starch pore surface, forming a fibrous cross-linked structure that disrupted the porous network of starch, resulting in the formation of larger network structures. Compared to the natural WMS group and the low TSP concentration group, the high TSP addition group exhibited more polysaccharide fiber cross-linking structures, suggesting a correlation between the polysaccharide fiber connectivity and the amount of polysaccharide added. The formation of these fibrous connections demonstrated the emergence of starch–polysaccharide interactions, leading to improved viscoelastic properties.

#### 3.1.2. FT-IR Analysis

**Figure 2:**
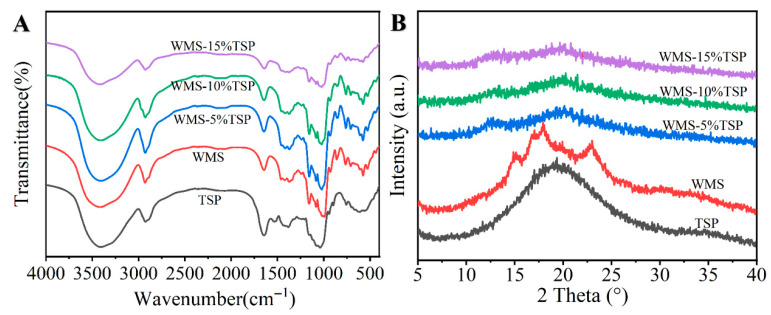
Physicochemical properties of waxy corn starch and WMS–TSP complexes. (**A**) FTIR spectra of TSP, native WMS, and WMS-TSP complexes containing 5%, 10%, and 15% TSP; (**B**) XRD patterns of TSP, native WMS, and WMS-TSP complexes containing 5%, 10%, and 15% TSP.

The structure of the WMS-TSP complex was investigated using FT-IR spectroscopy (Figure 2A). The infrared spectra of WMS and WMS-TSP complexes, within a concentration range of 5% to 15%, indicate an interaction between WMS and TSP. Compared to WMS, WMS-TSP system exhibited no detectable new absorption peaks arising from newly formed chemical bonds, suggesting that the interactions between WMS and TSP did not result in the formation of new functional groups. The additional bands observed at 1206 and 1700 cm^−1^ originate from characteristic functional groups inherent to TSP itself, and therefore represent spectral contributions from TSP rather than new peaks generated in the composite but instead relied on non-covalent interactions [23]. A prominent broad peak in the range of 3500–3200 cm^−1^ was attributed to O-H stretching vibrations. As the TSP concentration increased, the hydroxyl absorption peak exhibited a redshift and progressively widened, indicating an increase in hydrogen bonding [23]. The peak observed at 1645 cm^−1^ indicated the presence of O-H bonds from water molecules that are associated with the amorphous regions of starch. The observed decrease in the intensity of this peak upon TSP addition indicates a reduction in starch-bound water, reflecting changes in the water-binding environment within the WMS structure.

Notably, the modified WMS-TSP complex exhibited two additional characteristic peaks at 1206 cm^−1^ and 1700 cm^−1^ in the infrared spectrum. These bands were not newly generated but were already present in the FT-IR spectrum of TSP alone, reflecting the contribution of TSP functional groups within the composite. The absorption band at 1206 cm^−1^ was attributed to C–O–C asymmetric stretching of polysaccharide-associated groups, while the prominent peak at 1700 cm^−1^ corresponded to C=O stretching vibrations of carboxylic acid (–COOH) groups [24]. The absorbance ratios at 1047 cm^−1^/1022 cm^−1^ and 995 cm^−1^/1022 cm^−1^ were measured to assess the ordering and structural variations in the double helix of WMS under the influence of TSP. As indicated in Table 1, notable variations were observed across the samples. (*p* < 0.05). The absorbance ratios of the WMS-TSP complexes (c = 0, 5, 10, and 15%) showed two distinct trends with increasing TSP content: the R_1047_/_1022_ ratio exhibited a slight increase, whereas the R_995_/_1022_ ratio progressively decreased. This pattern indicates that TSP incorporation altered the local short-range molecular order of WMS rather than its crystalline structure, with the extent of structural modification becoming more evident at higher TSP levels. In addition, after pasting, the native granular morphology of WMS was no longer preserved; instead, the WMS-TSP systems developed gelatinized, freeze-dried porous composite networks, accompanied by changes in their internal short-range organization. Consequently, TSP reduced the short-range order in WMS.

#### 3.1.3. XRD Analysis

The crystallinity of starch plays a pivotal role in determining its physicochemical behavior, particularly digestibility, which is essential for both nutritional assessment and industrial applications [25]. As shown in Figure 2B, native WMS exhibited a typical A-type crystalline pattern, with characteristic diffraction peaks near 15°, 17°, 18°, and 23° (2θ). In contrast, the WMS-TSP samples showed nearly amorphous diffraction profiles, indicating that the native crystalline structure was almost completely lost during gelatinization and subsequent complex formation with TSP [10]. Although the overall peak intensities decreased across all TSP levels, no new diffraction peaks or crystalline polymorphs emerged, suggesting that TSP influenced only the weak residual short-range molecular order of the gelatinized starch rather than generating any new crystalline structures. Similar findings were reported by Zhou et al. [26], who observed that Laminaria japonica polysaccharides reduced starch crystallinity without altering its polymorphic class.

#### 3.1.4. TEM Analysis

**Figure 3:**
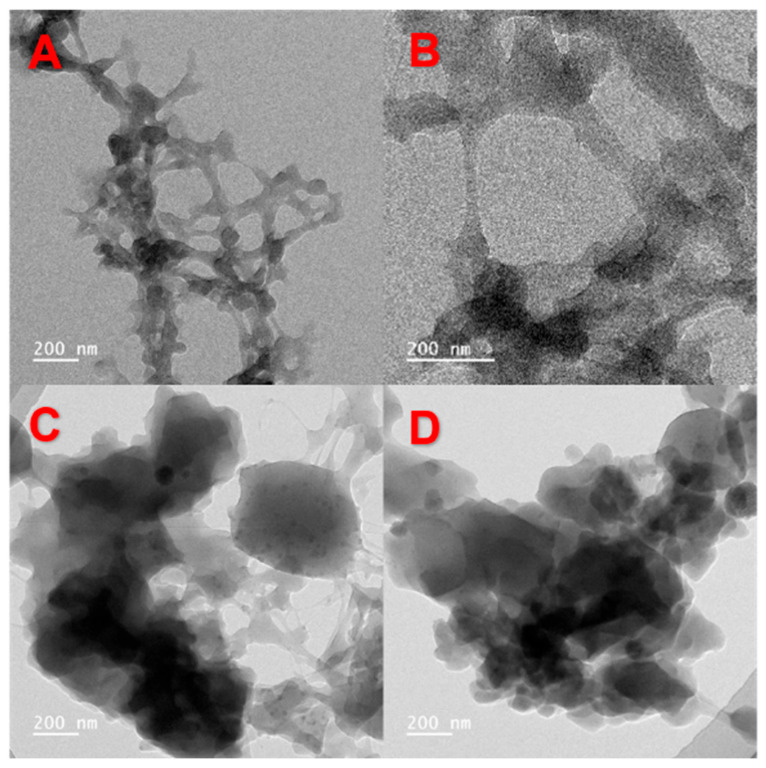
The TEM photomicrographs of waxy corn starch and WMS-TSP complexes (**A**) Waxy corn starch; (**B**) WMS-TSP complexes C = 5%; (**C**) WMS-TSP complexes C = 10%; (**D**) WMS-TSP complexes C = 15%.

To investigate the structural properties of WMS-TSP complexes, additional analysis was performed utilizing transmission electron microscopy. With the addition of TSP, the amylopectin in WMS gradually became attached to and was aggregated by TSP, as illustrated in Figure 3, forming macromolecular clusters. This aggregation was likely due to the hydrogen bonding between TSP and the starch molecular chains, which affected the aggregation of starch molecular chains during alcohol precipitation, consequently influencing the formation of complexes [26].

As the TSP content in the complex increased, the average particle size of WMS-TSP increased, thereby increasing the TSP content. The WMS-TSP complex exhibited the largest particle size (789.94 µm), with the particle size increasing as the TSP concentration in the WMS complex increased. This enlargement in hydrodynamic diameter was attributed to the interactions between WMS and TSP, which promoted aggregation or the formation of larger composite structures.

Transmission electron microscopy was used to study the morphological characteristics of WMS-TSP complexes (5% to 15%). The formation of WMS-TSP complexes produced complexes with diameters larger than bare WMS. Additionally, the degree of aggregation increased, resembling the structure of starch-polyphenol complexes.

#### 3.1.5. Particle Size

**Figure 4:**
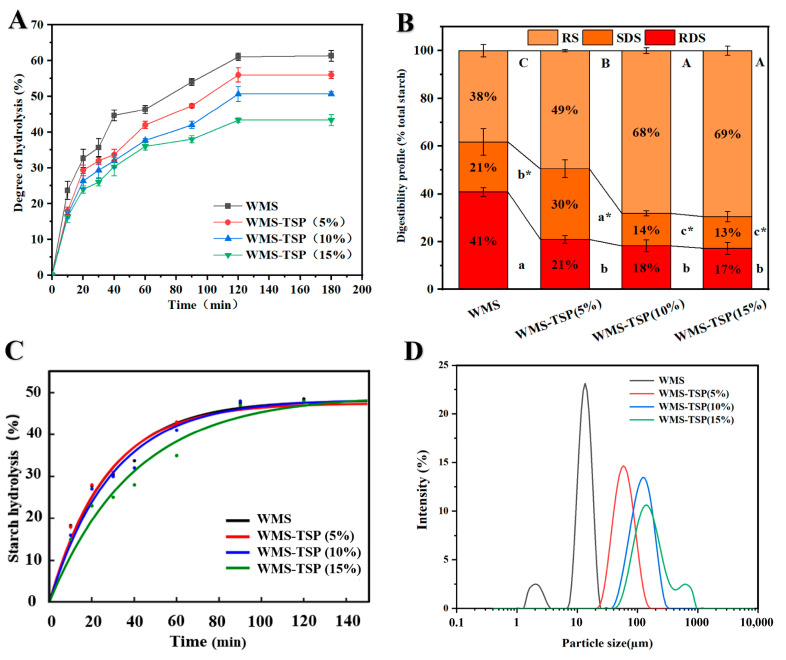
In Vitro digestibility and structural characteristics of WMS-TSP complexes. (**A**) Time-dependent starch hydrolysis of WMS and WMS-TSP samples. (**B**) Distribution of RDS, SDS, and RS fractions. Uppercase letters (**A**–**C**) indicate significant differences among samples for RS content (*p* < 0.05). Lowercase letters (a-c) indicate significant differences among samples for RDS content (*p* < 0.05). Asterisks (*) denote significant differences between WMS and WMS-TSP samples for SDS content (*p* < 0.05). (**C**) First-order kinetic fitting of hydrolysis curves. (**D**) Particle-size distribution of the corresponding complexes.

The particle size distribution of freeze-dried WMS and WMS-TSP is shown in Figure 4D. It can be seen that WMS-TSP (5%; 10%) both present a single peak distribution, with the main peaks located at 107.53 and 164.18 µm, respectively. Both WMS and WMS-TSP (15%) samples have two particle groups. The majority of the complexes are sized between 5–13 µm, while another group of complexes is smaller (2–3 µm).

Furthermore, Figure 4D shows the SEM images of WMS-TSP at different concentrations. The starch granules exhibited a bimodal size distribution, with the majority exceeding 5 μm in diameter and a minor fraction measuring below 3 μm, a finding that was consistent with the particle size distribution analysis. Additionally, WMS complexes exhibited diverse morphological characteristics, including spherical, ellipsoidal, cubic, and irregular geometries, with the majority demonstrating smooth surface textures; these observations align with previous reports in the literature [27].

In contrast, the WMS-TSP (15%) complexes are sized between 250–300 μm, while another group of complexes is larger (700 μm). The appearance of a bimodal distribution at high TSP concentrations is mainly due to the aggregation effect rather than the inherent characteristics of WMS-TSP having complexes of different sizes, further indicating that the presence of TSP affects the aggregation of starch complexes.

Compared with WMS-TSP, WMS exhibits a higher RDS content, rendering it more susceptible to enzymatic hydrolysis by digestive enzymes. This is consistent with the presence of pores on the surface of WMS complexes, which makes it easier for enzymes to enter the core of the complexes. Moreover, some of the WMS-TSP complexes show some indentations and overlapping honeycomb structures on their surfaces. Although these WMS-TSP complexes have similar particle sizes, the surface structure of the starch complexes is significantly related to digestibility.

#### 3.1.6. In Vitro Digestibility Analysis

The hydrolysis kinetics of WMS-TSP were characterized through in vitro simulation of gastrointestinal digestion processes, with the resultant degradation profile graphically presented in the accompanying Figure 4A. As shown in Figure 4A, the hydrolysis rate slows down over time, reaching a plateau at approximately 120 min. This is almost identical to the behavior observed in previous studies on native waxy corn starch, which also reached a plateau at 120 min [28]. For the WMS-TSP samples, the hydrolysis rate increased rapidly in the first 20 min, slowed down relatively between 20 and 120 min, and reached equilibrium after 120 min. Notably, TSP significantly reduced the digestibility of WMS in a concentration-dependent manner. The addition of 15% TSP resulted in a 17.70% reduction in WMS digestibility compared to the TSP-free control group.

Figure 4B depicts the digestibility profiles of WMS and WMS-TSP complexes. Native WMS consisted of 41% RDS, 21% SDS, and 38% RS, whereas TSP incorporation produced a pronounced shift in these fractions. The RDS content declined sharply to 21%, 18%, and 17% in WMS-TSP (5%), WMS-TSP (10%), and WMS-TSP (15%), corresponding to reductions of 20.1%, 23.1%, and 24.1%, respectively (*p* < 0.05). Concomitantly, the SDS and RS fractions increased substantially with increasing TSP levels, reaching up to 30% and 49% at 5% TSP and further rising to 14%/68% and 13%/69% in the 10% and 15% formulations. These results indicate that TSP effectively suppresses the formation of rapidly digestible starch while promoting more slowly digestible and enzyme-resistant fractions, thereby altering the overall hydrolysis kinetics of WMS. This trend is consistent with the findings of Wei et al., who also reported significant modulation of RDS and SDS fractions following TSP incorporation [29]. Although RS rose steadily with increasing TSP, the more pronounced decline in SDS at higher TSP levels resulted in a non-linear SDS + RS trend, with an initial increase at 5% TSP followed by stabilization or a slight decrease thereafter.

According to the literature, the decrease in starch digestibility caused by non-starch polysaccharides mainly stems from two mechanisms: The decreased digestibility of WMS-TSP samples is likely associated with enhanced polysaccharide starch interactions produced during co-heating [8,30]. The association between WMS and TSP promotes the formation of polysaccharide–starch complexes and induces structural rearrangements within the gelatinized matrix, thereby limiting the accessibility of α-amylase to internal substrates. Importantly, because TSP is a xyloglucan-type polysaccharide that is not hydrolyzed by α-amylase under the current in vitro digestion conditions, it functions solely as a non-digestible matrix component rather than contributing to the measured starch hydrolysis. The reduced hydrolysis rate may therefore be attributed to limited enzyme accessibility arising from TSP-induced structural constraints.

#### 3.1.7. Kinetic Analysis of In Vitro Starch Digestion

To further quantify the digestion behavior, the hydrolysis curves were fitted using a first-order kinetic model (LOS), *Ct* = *C∞*(1 *− e*^−^*ᵏᵗ*). The fitted equilibrium digestible fraction (C∞) did not differ markedly among samples and remained within a narrow range of 47.28–49.82% (Table 2), indicating that TSP addition had little impact on the final extent of starch hydrolysis. In contrast, the digestion rate constant (k) decreased significantly with increasing TSP concentration (*p* < 0.05). Native WMS exhibited the highest rate constant (0.0523 ± 0.0019 min^−1^), whereas WMS-TSP 5%, 10%, and 15% showed progressively lower values of 0.0403 ± 0.0023, 0.0338 ± 0.0035, and 0.0280 ± 0.0029 min^−1^, respectively. These trends were consistent with the fitted digestion curves (Figure 4C), which clearly demonstrate that TSP slowed the early-stage hydrolysis of WMS while maintaining a similar terminal hydrolysis level.

#### 3.1.8. Three-Phase Contact Angle

**Figure 5:**
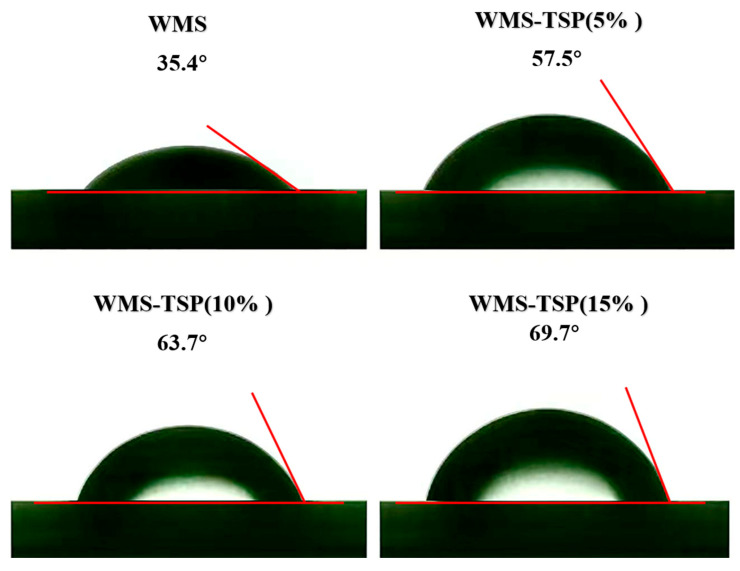
The three-phase contact angle of WMS and WMS-TSP complexes at different TSP addition levels.

The contact-angle results, as shown in Figure 5, reveal a clear shift in surface wettability once TSP is introduced. Native WMS displayed a θ value of 35.4°, reflecting its strong hydrophilicity, whereas samples containing TSP exhibited progressively larger angles of 57.5°, 63.7°, and 69.7°. The smooth, concentration-dependent rise suggests that TSP subtly reorganizes the surface microstructure—partly shielding hydrophilic regions and yielding a denser, less wettable interface. This behavior aligns well with trends reported in comparable starch–polysaccharide systems, reinforcing the role of TSP in tuning interfacial properties.

### 3.2. Characteristics of Emulsions Stabilized by WMS-TSP Complexes

#### 3.2.1. Physical Stability

**Figure 6:**
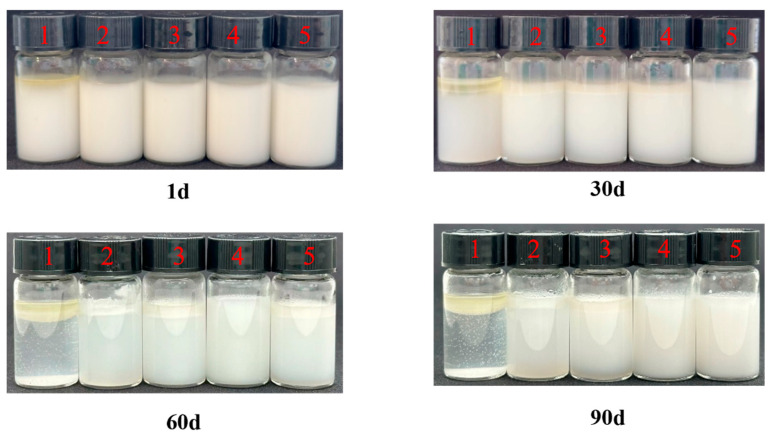
The stability of emulsions after 90 d of storage at 25 °C (1. WMS-TSP c = 0%; 2. WMS-TSP c = 0.5%; 3. WMS-TSP c = 1.0%; 4. WMS-TSP c = 1.5%; 5. WMS-TSP c = 2.0%).

The physical stability of emulsions prepared with different concentrations of WMS-TSP complexes is presented in Figure 6. Noticeable differences in stability were observed during the 90-day storage period. As shown in Table 3, the emulsification index (EI) of emulsions stabilized with 0.5% and 1.0% WMS-TSP did not differ significantly (*p* > 0.05), whereas the formulation containing 1.5% WMS-TSP exhibited the highest EI value. The visual appearance of the emulsions was consistent with the EI results, with the 1.5% WMS-TSP emulsion showing the greatest resistance to phase separation over time. These observations indicate that increasing the concentration of WMS-TSP enhances its emulsifying ability, as reflected by the higher EI values, thereby improving the long-term physical stability of the emulsion system. Emulsions stabilized with WMS-TSP complexes exhibited a clear concentration-dependent change in droplet size distribution and zeta potential (Table 4). As TSP concentration increased from 0.5% to 2.0%, the characteristic diameters D10, D50, D90, and Dav all increased significantly (*p* < 0.05), indicating progressive droplet growth due to enhanced particle adsorption at the interface. In particular, the Dav value increased from 136.47 μm (0.5% TSP) to 175.94 μm (2.0% TSP), demonstrating a substantial enlargement of emulsion droplets.

Meanwhile, the absolute zeta potential values also increased from −39.63 mV to −56.41 mV with increasing TSP concentration, reflecting stronger electrostatic repulsion between droplets. The simultaneous enlargement of droplets and increase in the magnitude of surface charge suggests that TSP effectively enhanced the interfacial adsorption capacity of WMS-TSP complexes, leading to improved electrostatic stabilization of the emulsion system.

#### 3.2.2. Rheological Analysis

**Figure 7:**
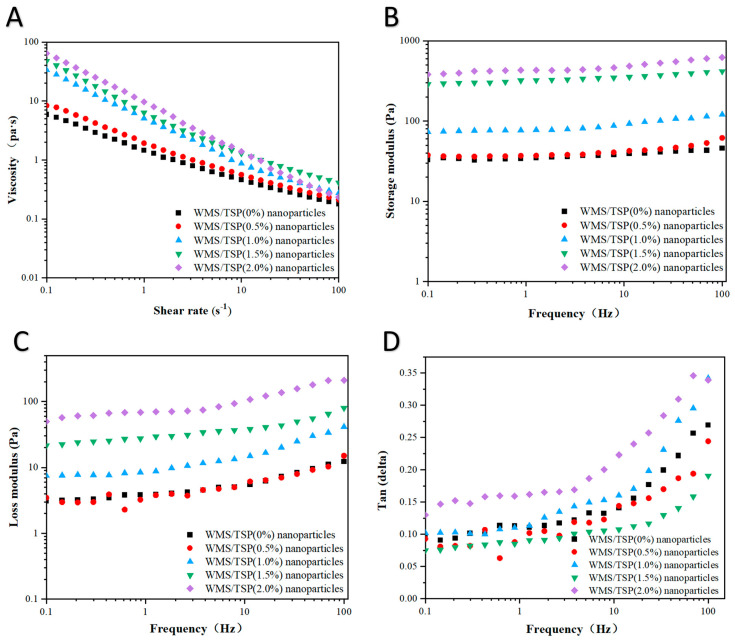
(**A**–**D**) The rheological properties of emulsions with different concentrations of WMS-TSP complexes were investigated.

The rheological properties of emulsions provide us with important insights into the mechanical network structure within these emulsions, which are crucial for evaluating their stability and functionality. In this study, we investigated the amplitude sweep, frequency sweep, and shear rate sweep behaviors of emulsions stabilized by WMS-TSP complexes were examined, with varying concentrations of TSP incorporated, as depicted in Figure 7. As shown in Figure 7B, both the storage modulus (G′) and the loss modulus (G″) increased as the WMS-TSP concentration increased from 0% to 2%, indicating a progressive enhancement in the viscoelastic strength of the emulsions. For all formulations, G′ remained higher than G″ throughout the measured frequency range, suggesting that the emulsions behaved predominantly as weak elastic networks, which is consistent with previous observations for concentrated emulsion systems [31]. Such weak gel-like behavior may arise from the increased droplet packing and inter-droplet interactions at higher WMS-TSP levels [32]. As shown in Figure 7C, both moduli increased with frequency; however, the rate of increase in G″ was greater than that of G′, implying that the viscous response became more prominent at higher oscillation frequencies [33]. Furthermore, all emulsions exhibited shear-thinning behavior, as evidenced by the steady decrease in apparent viscosity with increasing shear rate (Figure 7D), a typical characteristic of structured emulsions.

#### 3.2.3. Confocal Laser Scanning Microscopy (CLSM)

**Figure 8:**
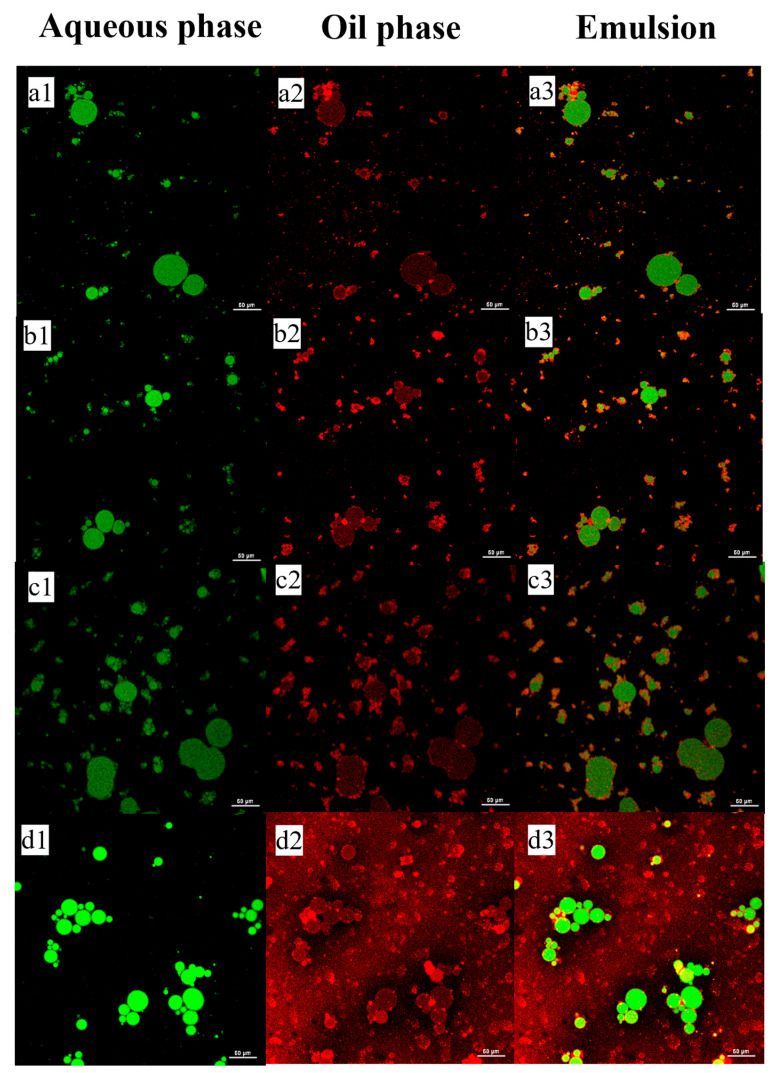
Confocal laser scanning micrographs of WMS-TSP-stabilized O/W emulsions. The aqueous phase was stained with Nile Blue A and appears in green, while the oil phase was stained with Nile Red and appears in red. (**a1**–**d1**) Aqueous phase stained with Nile Blue A (green fluorescence); (**a2**–**d2**) Oil phase stained with Nile Red (red fluorescence); (**a3**–**d3**) Merged images showing the distribution of the aqueous and oil phases in the emulsions at different TSP concentrations.

To elucidate the arrangement of WMS-TSP complexes at the interface, the microstructure was examined using CLSM. The images demonstrated that WMS-TSP complexes were densely distributed around the oil droplets, forming a network-like structure. These oil droplets were encapsulated within a three-dimensional network, which acted as a mechanical barrier to inhibit droplet coalescence. Furthermore, the addition of starch granules enhances the viscosity of the continuous phase, thereby improving the physical stability of the emulsion. The CLSM results were consistent with previously reported stabilization mechanisms [34]. As the concentration of WMS-TSP increased, the CLSM images (Figure 8) showed more uniformly dispersed oil droplets and a thicker continuous matrix surrounding them. The denser polysaccharide network likely enhanced droplet confinement and reduced droplet–droplet contact, thereby limiting coalescence and contributing to improved emulsion stability. However, at a concentration of 2%, the CLSM images (Figure 8(d1–d3)) showed more pronounced droplet association and the appearance of larger droplet domains compared with the lower-concentration samples. These microstructural features suggest that densely packed WMS-TSP networks may alter droplet organization at elevated concentrations. Such changes may reflect increased intermolecular interactions among starch polysaccharide chains, including hydrogen bonding and hydrophobic associations. This observation aligns with the EI measurements, which plateaued between 1.5% and 2%, implying that further increases in WMS-TSP concentration did not translate into additional gains in macroscopic stability.

## 4. Conclusions

This study investigated how the incorporation of TSP modified the structural and functional properties of WMS, and systematically evaluated its effects on digestibility and emulsion stabilization. The SEM images showed that although the native granular morphology of WMS was completely lost after pasting, the gelatinized WMS formed porous, aggregated networks after TSP addition. FT-IR results revealed changes in hydrogen-bonding patterns and short-range molecular order, as reflected by decreased 1047/1022 cm^−1^ ratios and increased 1022/995 cm^−1^ ratios. XRD analysis showed nearly amorphous profiles, with a further reduction in residual short-range order after TSP addition. The digestion results showed a pronounced reduction in rapidly digestible starch accompanied by a lower hydrolysis rate constant and higher proportions of SDS and RS, indicating that TSP slowed the progression of enzymatic hydrolysis. Emulsions stabilized with WMS-TSP complexes displayed increased droplet charge, smaller and more uniform droplet structures, and improved long-term physical stability. CLSM micrographs showed that WMS-TSP complexes adsorbed at the oil–water interface and formed compact interfacial networks that effectively limited droplet coalescence. Collectively, these findings show that TSP can effectively modify WMS to create clean-label starch–polysaccharide systems with controlled digestibility and improved emulsion stability. In addition, the resulting WMS-TSP complex presents practical potential as a natural stabilizer for foods that require enhanced emulsion stability or regulated starch digestion.

## Figures and Tables

**Table 1:** FTIR structural parameters, relative crystallinity, and volume-based particle size characteristics (D_10_, D_50_, D_90_) of WMS and WMS-TSP complexes.

Sample	R_1047/1022_	R_1022/955_	Relative Crystallinity (%)	D_10_ (μm)	D_50_ (μm)	D_90_ (μm)
WMS	0.895 ± 0.006 ^a^	1.24 ± 0.0091 ^d^	26.90 ± 0.02 ^a^	4.85 ± 1.40 ^a^	8.72 ± 0.11 ^a^	11.70 ± 1.62 ^a^
WMS-TSP (5%)	0.905 ± 0.002 ^b^	0.95 ± 0.0033 ^c^	3.21 ± 0.09 ^b^	68.06 ± 2.18 ^b^	105.71 ± 0.61 ^b^	164.18 ± 0.31 ^b^
WMS-TSP (10%)	0.917 ± 0.012 ^c^	0.83 ± 0.0032 ^b^	4.57 ± 0.19 ^c^	91.28 ± 1.53 ^c^	164.18 ± 0.15 ^c^	255.00 ± 0.05 ^c^
WMS-TSP (15%)	0.928 ± 0.001 ^d^	0.55 ± 0.0031 ^a^	4.54 ± 0.13 ^c^	141.77 ± 2.91 ^d^	295.31 ± 0.42 ^d^	824.99 ± 1.31 ^d^

D10 and D90, 10 and 90% (*v*/*v*) of particles are smaller than this size; D50, median particle diameter; Dav, average size; Mean of 3 determinations ± standard deviation; Values with the different letter in the same column are significantly different (*p* < 0.05).

**Table 2:** Kinetic parameters of in vitro starch digestion fitted by the LOS.

Sample	C∞ (%)	k (min^−1^)	R^2^
WMS	49.82 ± 0.94	0.0523 ± 0.0019 a	0.979
WMS-TSP 5%	47.28 ± 0.14	0.0403 ± 0.0023 b	0.979
WMS-TSP 10%	47.99 ± 0.73	0.0338 ± 0.0035 b	0.977
WMS-TSP 15%	48.73 ± 1.19	0.0280 ± 0.0029 b	0.971

Values are expressed as mean ± SD (*n* = 3). Different lowercase letters within a column indicate significant differences at *p* < 0.05.

**Table 3:** Effect of WMS-TSP complex concentrations on the emulsification index of emulsions.

Sample	Emulsifying Index (EI)			
	Day 1	Day 30	Day 60	Day 90
WMS	-	-	-	-
WMS-TSP (0.5%)	84.7 ± 1.8 ^c^	75.3 ± 3.1 ^c^	74.0 ± 3.5 ^b^	70.7 ± 2.3 ^c^
WMS-TSP (1.0%)	88.7 ± 3.1 ^bc^	78.0 ± 5.3 ^bc^	75.3 ± 5.0 ^b^	74.7 ± 4.2 ^bc^
WMS-TSP (1.5%)	93.3 ± 2.3 ^a^	90.7 ± 1.2 ^a^	90.3 ± 1.1 ^a^	89.3 ± 1.2 ^a^
WMS-TSP (2.0%)	91.3 ± 1.7 ^a^	82.7 ± 1.0 ^b^	80.3 ± 2.1 ^b^	77.3 ± 1.9 ^b^

Mean of 3 determinations ± standard deviation; Values with the different letter in the same column are significantly different (*p* < 0.05).

**Table 4:** Droplet size distribution and zeta potential of oil-in-water emulsions stabilized by WMS-TSP complexes.

Sample	Droplet Size Distribution (μm)				Zeta Potential (mV)
	D10	D50	D90	Dav	
WMS	-	-	-	-	-
WMS-TSP (0.5%)	110.06 ± 1.40 ^a^	133.38 ± 0.19 ^a^	176.89 ± 1.31 ^a^	136.47 ± 0.47 ^a^	−39.63 ± 2.66 ^c^
WMS-TSP (1.0%)	117.43 ± 0.18 ^a^	152.17 ± 0.32 ^b^	186.97 ± 0.47 ^b^	154.14 ± 0.68 ^b^	−47.96 ± 1.48 ^b^
WMS-TSP (1.5%)	131.87 ± 1.13 ^b^	174.31 ± 0.61 ^c^	201.84 ± 0.73 ^c^	179.66 ± 0.39 ^c^	−52.01 ± 0.37 ^a^
WMS-TSP (2.0%)	127.15 ± 0.78 ^c^	169.29 ± 0.41 ^c^	198.67 ± 0.89 ^c^	175.94 ± 1.05 ^c^	−56.41 ± 1.14 ^a^

D10 and D90, 10 and 90% (*v*/*v*) of particles are smaller than this size; D50, median particle diameter; Dav, average size; Mean of 3 determinations ± standard deviation; Values with the different letter in the same column are significantly different (*p* < 0.05).

## Data Availability

The data presented in this study are available on request from the corresponding author.

## Supplementary Materials

The following supporting information can be downloaded at: https://www.mdpi.com/article/10.3390/foods14234152/s1, Figure S1: The binding mode of sugar unit and the composition and molecular model of chain repeat unit of Tamarind polysaccharide (Zhang et al., 2020) [35].
