# Supplementary material for: Exploring the Role of Tamarind Seed Polysaccharides in Modulating the Structural, Digestive, and Emulsion Stability Properties of Waxy Corn Starch Composites
Source: Foods. 2025 Dec 3;14(23):4152. doi: 10.3390/foods14234152 (PMC12692401; doi:10.3390/foods14234152)
Supplement: Supplementary file 1 [file foods-14-04152-s001.zip › foods-3989237-supplementary.pdf]

Supporting Information

For

Exploring the Role of Tamarind Seed Polysaccharides in Modulating the Structural, Digestive, and Emulsion Stability Properties of Waxy Corn Starch Composites

Xiangyu Ya[a,b,c,d],Yongshuai Ma[a,b,c,d], Zibo Song[e,f], Yongli Jiang[a,b,c,d],Chaofan Guo[a,b,c,d], Junjie Yi[a,b,c,d] *

[a] Faculty of Food Science and Engineering, Kunming University of Science and Technology, 650500 Kunming, Yunnan, China

[b] Key Laboratory of Plateau Characteristic Prepared Food in Yunnan Province, Kunming 650500, China

[c] International Green Food Processing Research and Development Center of Kunming City, 650500 Kunming, Yunnan, China

[d] Yunnan Engineering Research Center for Fruit & Vegetable Products, 650500 Kunming, Yunnan, China

[e] Yunnan Maoduoli Key Laboratory of Applied Technology for Special Forest Fruits, 653100 Yuxi, Yunnan, China

[f] Yunnan Maoduoli Group Food Co., Ltd., 653100 Yuxi, Yunnan, China

*Corresponding author Junjie Yi

E-mail addresses: yaxiyu84@gmail.com (X.Ya), mays186@163.com (Y.Ma), 2945893036@qq.com (Z.Song), yongli_jiang0617@163.com (Y. Jiang), guochaofnafan@outlppk.com (C.Guo), junjieyi@kust.edu.cn (J. Yi)

Supplementary Figures

Figure S1:

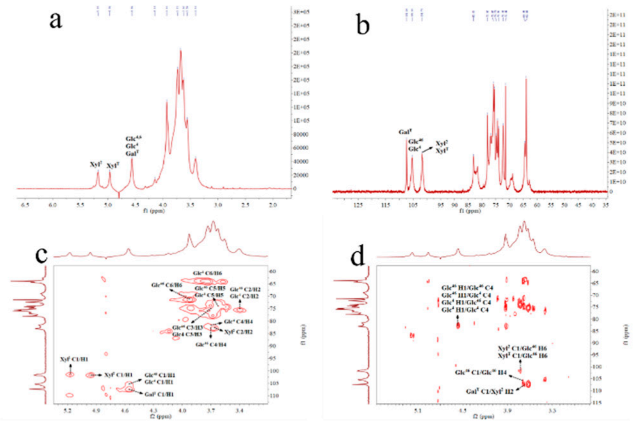

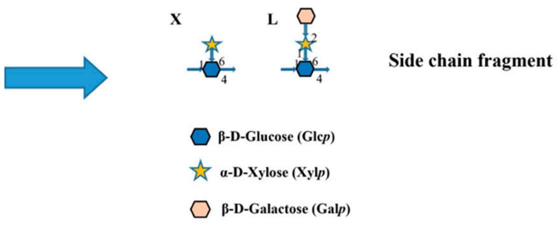

NMR spectroscopy of TSP (a:^{1}H NMR, b:^{13}C NMR, c:HSQC, d:HMBC)

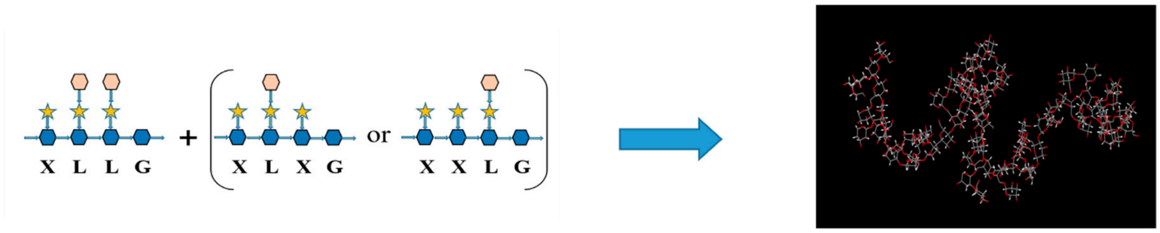

Chain model of TSPS (molecular weight about 14 kDa) with polymerization degree of 10 repeat units in water system

Figure S1: The binding mode of sugar unit and the composition and molecular model of chain repeat unit of Tamarind polysaccharide (Zhang, et al.2020) [1].

The TSP chain consisted of unsubstituted β-D-Glcp (G), monosubstituted X and dissubstituted L fragments connected by 1,4→ glycosidic bond, and the backbone was β-(1→4) –glucan.

TSP is a typical xyloglucan with Mw of 524.0 kDa. The backbone were highly substituted by xylose and galactose, and the high content of galactose induced the chain to form a compact and regular structure.

References

1. Zhang H, Zhao T, Wang J, et al. An amendment to the fine structure of galactoxyloglucan from Tamarind (Tamarindus indica L.) seed. *Int. J. Biol. Macromol.*, **2020**, 149: 1189-1197. https://doi.org/10.1016/j.ijbiomac.2020.01.284
